# MpBsmi: A new algorithm for the recognition of continuous biological sequence pattern based on index structure

**DOI:** 10.1371/journal.pone.0195601

**Published:** 2018-04-23

**Authors:** Weina Li, Jiadong Ren

**Affiliations:** 1 College of Information Science and Engineering, Yanshan University, Qinhuangdao, Hebei P.R.China, 066004; 2 The Key Laboratory for Computer Virtual Technology and System Integration of Hebei Province, Qinhuangdao City, P.R.China, 066004; 3 The Key Laboratory for Software Engineering of Hebei Province, Qinhuangdao City, P.R.China, 066004; Northeast Normal University, CHINA

## Abstract

A significant approach for the discovery of biological regulatory rules of genes, protein and their inheritance relationships is the extraction of meaningful patterns from biological sequence data. The existing algorithms of sequence pattern discovery, like MSPM and FBSB, suffice their low efficiency and accuracy. In order to deal with this issue, this paper presents a new algorithm for biological sequence pattern mining abbreviated MpBsmi based on the data index structure. The MpBsmi algorithm employs a sequence position table abbreviated ST and a sequence database index structure named DB-Index for data storing, mining and pattern expansion. The ST and DB-Index of single items are firstly obtained through scanning sequence database once. Then a new algorithm for fast support counting is developed to mine the table ST to identify the frequent single items. Based on a connection strategy, the frequent patterns are expanded and the expanded table ST is updated by scanning the DB-Index. The fast support counting algorithm is used for obtaining the frequent expansion patterns. Finally, a new pruning technique is developed for extended pattern to avoid the generation of unnecessarily large number of candidate patterns. The experiments results on multiple classical protein sequences from the Pfam database validate the performance of the proposed algorithm including the accuracy, stability and scalability. It is showed that the proposed algorithm has achieved the better space efficiency, stability and scalability comparing with MSPM, FBSB which are the two main algorithms for biological sequence mining.

## Introduction

Biological sequence is an important component of bioinformatics data, generally including three categories: DNA sequence, RNA sequence and protein sequence [1]. Since the human genome project was completed in 2003, we have seen an explosive growth in bioinformatics data. By April 2017, there are 200877884 sequences in the GenBank [2] database. Since the release in 1982, the base number in GenBank has doubled by about every 18 months. Sequential pattern mining is an important method used for discovering frequent patterns and association rules in the data mining field [3], providing an effective way to find important rules of biological sequences. Biological sequence patterns can be used to predict human diseases and provide evidences for artificial nucleotides, artificial proteins and so on. Biological sequence pattern mining has become a hot research direction in recent years [4].

Frequent pattern mining is an important part of sequential pattern mining. Frequent pattern mining algorithms [5] contain two main categories, the Apriori based algorithm and the FP-growth based algorithm. The Apriori algorithm proposed by Agrawal et al [6] is the most commonly used in the association rule discovery. The frequent pattern mining has appeared in a variety of applications such as sequence pattern mining [7], structural mining [8], classification association mining [9], frequent pattern based on clustering [10] and so on. A number of sequential pattern mining algorithms have been proposed during the past years, such as incremental mining [11], top-k sequence pattern mining [12], maximum sequence pattern mining [13], constraint sequence pattern [14], weighted sequence pattern mining [15], closed sequence pattern mining [16–17]. To improve the efficiency and accuracy Oza K et al. [18] proposed an algorithm for regular expression constraint, weight constraint and length constraint to solve the problems of user interests, optimization for support threshold and accuracy. Xue F et al. [19] improved the PrefixSpan algorithm [20], and then proposed PrefixSpan-x to reduce unnecessary memory usage. Kemmar A et al. [21] proposed a top-k sequence pattern algorithm based on prefix projection and global constraints. This global constraint can take into account the quantity, item relation and regular expression and so on easily.

The classical sequence pattern algorithm is the footstone of biological sequence mining. The current sequence pattern algorithms are mainly improved from efficiency and precision. With the improvement of computer hardware performance, the algorithms with high memory utilization and changing time with space emerge. Lin et al. [22] proposed a fast sequence pattern algorithm named MEMISP based on memory index. This algorithm only needs scanning the sequence database once. An index table structure is proposed to record the positions of subsequence in the sequence and obtain the longer sequence pattern gradually in a recursive way. Its efficiency is higher than PrefixSpan and GSP algorithm. Then Ren et al. [23] proposed a closed sequential pattern mining algorithm MIWCSpan based on the memory index and item weights, considering the time interval of items and avoiding scanning database multiple times. Zeng et al. [24] proposed an algorithm based on the time interval weight and memory index, which further indicates the importance of time interval weight and improves the utilization of memory. Ren et al. [25] proposed an algorithm named MIFSPM, which uses the memory index structure of the frequent pattern tree. Each node in the tree stores the index table instead of storing the frequent pattern. This algorithm uses two kinds of support threshold constraint and sets the support threshold according to users’ interaction.

In recent years, extensive research has been conducted on the biological sequence pattern mining focusing on the improvement of efficiency and precision. Yun [26] proposed an algorithm based on the prefix projection named BioPM, which can effectively mine the consensus sequences in the protein sequences. Chen et al. [27] proposed an algorithm MSPM based on the prefix tree and pattern expansion method. This algorithm abandons a large number of non-relevant candidate patterns, avoiding generating a large number of candidate projection sequences and many short candidate patterns which cost a lot of memory and time in dealing with complex biological sequences. Wang et al. [28] proposed an algorithm FBSB based on the bitmap storage structure and a fast sorted list. It avoids generating candidate patterns.

Parallel computing technology has also been studied for the sequence data mining [29]. Jiang et al. [30] used the Spark framework to mine sequence patterns by dealing with the uncertainty of DNA data. M Klein et al. [31] applied the Hadoop and Spark frameworks in their proposed biospark framework to deal with large data sets of biological sequences. Talouki MS et al. [32] conducted an algorithm based on a parallel prefix tree in handling protein data over several computers on a LAN network. The algorithm adopts the constraint of dynamic task assignment and selection sampling technology to avoid the machine idling and improve the precision.

The current biological sequence mining algorithms were generally focused on improvement of the classical sequence pattern algorithms or use the parallel computing. The existing sequence pattern algorithms suffer from the low efficiency, high memory utilization and low precision. To address these issues, this paper presents a biological sequence pattern mining algorithm MpBsmi based on the data index technology. This new algorithm has the following features:

(1)A sequence position table ST and a sequence database index structure DB-Index are employed to improve the efficiency in scanning database and pattern support counting.

(2)A method is proposed to do the sequence pattern expansion easily through the position table and data index.

(3)A fast support counting method getting continuous sequence patterns is put forward.

(4)A novel method to prune the extended pattern is presented, which can filter out the subsequences whose support count is less than the threshold by index database, reducing the generation of a lot of candidate patterns.

Remaining of the paper is organized as follows. In Section 2, problems are defined, and the model is built. Section 3 presents our proposed algorithms including the position table, fast support counting algorithm and the updating algorithm of the position table and data index. Section 4 conducts the experiments and evaluate the performance. The discussions are made in Section 5. The paper is concluded in Section 6.

## Definition and model

Biological sequences typically include DNA, RNA and protein sequences. They are different from general transaction sequences. DNA is a long chain polymer with four types of deoxy nucleotide: adenine (dAMP), thymine (dTMP), cytosine(dCMP), and guanine cytosine (dGMP). RNA is the ribonucleic acid which is a chain molecule formed by condensing two phosphate ester by DNA. RNA is composed of phosphoric acid, ribose and base. The bases of RNA are mainly in four kinds, i.e. adenine (A), guanine (G), cytosine (C) and uracil (U), where uracil (U) replaces thymine (T) in DNA. Proteins are formed by RNA translation and are large molecules containing 20 amino acid residues. In this chapter, formal concepts of biological sequence are given followed by the definitions and algorithm model.

### Preliminary concepts

DNA sequence. There exists an alphabet set ∝ = {A,C,G,T}, the DNA sequence can be represented by DS = <S_1_*S*_2_*S*_3_⋯S_*n*_>, where n∈Z^+^, S_*n*_∈∝, S_*n*_ is called an item.

RNA sequence. There exists an alphabet set *τ* = {A,C,G,U}, the RNA sequence can be represented by RS = <S_1_S_2_S_3_⋯S_*x*_>, where x∈Z^+^, S_*x*_∈*τ*, S_*x*_ is called an item.

Protein sequence. There exists an alphabet set *ω* = {A,C,D,E,F,G,H,I,K,L,M,N,P,Q,R,S,T,V,W,Y} of 20 different letters, the DNA sequence can be represented by PS = <S_1_S_2_S_3_⋯S_*m*_>, where m∈Z^+^, S_*m*_∈*ω*, S_*m*_ is called an item.

Biological sequence. Given a sequence BS, if the type of BS, BS.type∈{DS,RS,PS}, then BS is called a biological sequence. The DS, RS and PS are denoted as types of the BS. For a type of BS as DS, it can be written as BS.type = [DS].

Parent sequence and child sequence. Given any two sequences BS_1_ = <S_11_S_12_S_13_⋯S_1*m*_> and BS_2_ = <S_21_S_22_S_23_⋯S_2*n*_>, and the conditions of BS_1_.type = BS_2_.type, m, n∈Z^+^ and m≤n are satisfied. If S_11_ = S_21_, S_12_ = S_22_,⋯,S_1*M*_ = S_2*M*_, then BS_1_ is the child sequence of BS_2_ and BS_2_ is the father sequence of BS_1_. That is to say BS_2_ contains BS_1_, denoted as BS_1_⊆BS_2_.

Example 1: Assuming a set *ω* = {A, C, D, E, F, G, H, I, K, L} of 10 different letters, the sequence BS_1_ = <ACDEFGHIKL> can represent a protein sequence. If BS_2_ = <ACDEFG>, then BS_2_⊆BS_1_. Table 1 shows four sequences in the protein family indexed by PF00106 in the sequence database Pfam with the version 31.0 [33]. Length of biological sequence. Given a biological sequence BS_1_, the number of items contained in BS_1_ is called the length of BS_1_ which is written as |BS_1_|.

**Table 1:** An example of the biological sequence.

index	sequence
1	KITIITGGTRGIGFAAAKLFIENGAKVSIFGETQEEVDTALAQLKELYPE EEVLGFAPDLTSRDAVMAAVGTVAQKYGRLDVMINNAGITMNSVFSRVS EEDFKNIMDINVNGVFNGAWSAYQCMKDAKQGVIINTASVTGIYGSLSG IGYPTSKAGVIGLTHGLGREIIRKNIRVVGVAPGVVDTDMTKGLPPEIL
2	EVALVTGATSGIGLEIARRLGKEGLRVFVCARGEEGLRTTLKELREAGVE ADGRTCDVRSVPEIEALVAAVVERYGPVDVLVNNAGRPGGGATAELADEL WLDVVETNLTGVFRVTKQVLKAGGMLERGTGRIVNIASTGGKQGVVHAA PYSASKHGVVGFTKALGLELARTGITVNAVCPGFVETPMAASVREHYS
3	RVALVTGATSGIGLATARLLAAQGHLVFLGARTESDVIATVKALRNDGLEA EGQVLDVRDGASVTAFVQAAVDRYGRIDVLVNNAGRSGGGVTADLTDEL WDDVIDTNLNSVFRMTRAVLTTGGMRTRERGRIINVASTAGKQGVVLGAP YSASKHGVVGFTKALGNELAPTGITVNAVCPGYVETPMAQRVRQGYA
4	PVALVTGATSGIGLAIARRLAALGARTFLCARDEERLAQTVKELRGEGF DVDGTVCDVADPAQIRAYVAAAVQRYGTVDILVNNAGRSGGGATAEIADE LWLDVITTNLTSVFLMTKEVLNAGGMLAKKRGRIINIASTGGKQGVVHAV PYSASKHGVVGLTKALGLELARTGITVNAVCPGFVETPMAERVREHYA

Biological sequence database. The database of biological sequence is a warehouse of biological sequences. It can be represented as BS_db = {BS_1_, BS_2_,⋯,BS_*n*_}, where BS_db[n] = BS_*n*_ and n∈Z^+^.

Length of BS_db. The length of BS_db is the number of sequences it contains, expressed as |BS_db|.

### Definitions

Sequence position table and index database are two main data structures employed in this paper. Sequence position table records the locations of all the subsequences that appear in the sequences. The index database records all the indexes of the subsequences.

Definition 1. Continuous biological sequence CBS. Given two biological sequences BS_1_ = <S_11_S_12_S_13_⋯S_1*m*_> and BS_2_ = <S_21_S_22_S_23_⋯S_2*n*_>, if S_21_ = S_1*y*_, S_2(1+1)_ = S_1(*y*+1)_,⋯, S_2*n*_ = S_1(*y*+*d*)_, and 1≤y,y+d≤m,y,d,n,m∈Z^+^, then BS_2_ is called a continuous biological sequence CBS of BS_1_.

Definition 2. Subsequence. Given BS_1_ = <S_11_S_12_S_13_⋯S_1*m*_> and BS_2_ = <S_21_S_22_S_23_⋯S_2*n*_> are two biological sequences, if there exists S_2*x*_ = S_1*y*_, S_2(*x*+1)_ = S_1(*y*+1)_,⋯,S_2*m*_ = S_1*m*_, and 1≤x,1≤y,x≤m≤n,y≤m≤_*n*_,x,y, n, m∈Z^+^, then BS_1_ is called a subsequence of BS_2_, expressed as BS_1_⊙BS_2_.

Wherein x refers to the position of BS_1_ appearing in BS_2_, expressed as pos = x. Then the collection of all the positions of BS_1_ appearing in BS_2_ is represented as POS(BS_1_⊙BS_2_) = {x|x = pos,pos≤|BS_2_|, pos∈Z^+^, BS_1_⊙BS_2_}.

Definition 3. Sequence position table ST. ST is a two-dimensional table that records all the positions where the subsequences appear in the parent sequences. A row in the position table represents a subsequence and its positions in the sequence database. ST can be expressed as ST = {ST(S)|S∈BS_db};

Wherein the position table of the sequence S is denoted as ST(S) = [SP_(*s*)_1_, SP_(*s*)_2_,⋯,SP_(*s*)_*n*_], where n∈Z^+^, SP_(*s*)_*n*_ represents a list of positions for the sequence S in the n^*th*^ sequence. SP_(*s*)_*n*_ = {x, y, z, …, m}, where x<y<z<⋯<m<|BS_*n*_|, x, y, z,⋯, m∈Z^+^. Also it can be expressed as ST(S)[n] = SP_(*s*)_*n*_. *ST(S)[n]*.*pos*. The pos represents the position of sequence S contained in the n^*th*^ sequence. The length of SP_(*s*)_*n*_ is the number of positions it contains, expressed as |SP_(*s*)_*n*_|.

If every length of sequences in the position table ST is k, the position table is also called k-ST.

Example 2. Table 2 shows an example of a sequence database. From the table, we can get the items a, b, c contained in the sequences. In the first sequence, <abc> is a continuous biological sequence CBS and a subsequence of the first sequence. A part of ST for the single items is shown in Table 3. As can be obtained from Table 3, ST(a) = [{1,5}, {1,5}, {4}, {1,4}], wherein SP(a)_1 = {1,5}, item a appears in the first and fifth position in the first sequence.

**Table 2:** Instances of sequence database.

Serialnumber	sequence
1	abcbac
2	acbcab
3	bcbabc
4	acbabc

**Table 3:** Instances of position table.

item	location information
*a*	[{1,5}, {1,5}, {4}, {1,4}]
*b*	[{2,4}, {3,6}, {1,3,5}, {3,5}]
*c*	[{3,6}, {2,4}, {2,6}, {2,6}]

Definition 4. Sequence database index DB-Index. Given a sequence database BS_db = {BS_1_, BS_2_,⋯BS_*n*_}, n∈Z^+^, if the subsequence a is contained by the sequences in the BS_db, then the database index of subsequence a can be expressed as DB-Index(a) = {m|BS_*m*_∈BS_db, a∈BS_*m*_, m∈Z^+^}, where m is the index number of the sequences that contains the subsequence a.

DB-Index contains the indexes of all the subsequences, expressed as DB-Index = {DB-Index(a)|BS_*m*_∈BS_db, a∈BS_*m*_, m∈Z^+^}.

The times the index numbers containing subsequence a appear in DB-Index(a) is called the length of the sequence database index of subsequence a, expressed as |DB-Index(a)|. If every length of the sequences in the DB-Index is k, the DB-Index is also called k-DB-Index.

Definition 5. Biological sequence pattern BSP. Given a minimum support threshold minsup, a sequence database BS_db, a sequence BS_1_ and BS_1_ is a CBS, the support of BS_1_ can be expressed as Support (BS_1_) = |DB-Index(BS_1_)|/|BS_db|. If Support (BS_1_)≥minsup, BS_1_ is called a biological sequence pattern. |DB-Index(BS_1_)| is also known as the support count of the sequence BS_1_, expressed as Support (BS_1_).

If the length of a BSP is l, the pattern is also called l-BSP.

Property 1. If *ρ* is equal to the number of the non-empty SP_(*BS*)_ in ST(BS), then |DB-Index(BS)| = *ρ*.

Proof. Let a sequence database BS_db = {BS_1_, BS_2_,⋯,BS_*n*_}, n∈Z^+^. Since SP_(*BS*_*i*)_≠*ϕ*, i∈Z^+^, BS⊙BS_*i*_ and i∈DB-Index(BS), *ρ* is equal to the number of i that meets the conditions. Therefore |DB-Index(BS)| = *ρ*.

Example 3. As the sequence database in Table 2, DB-Index of item a can be expressed as DB-Index(a) = {1,2,3,4}. The DB-Index of the sequence <ab> can be written as DB-Index(ab) = {1,2,3,4}. Suppose minsup = 50%, |DB-Index(a)| = 4, |BS_db| = 4, 4/4 = 100%, and the sequence <ab> is a CBS, then <ab> is a BSP.

### Algorithm model

**Fig 1:**
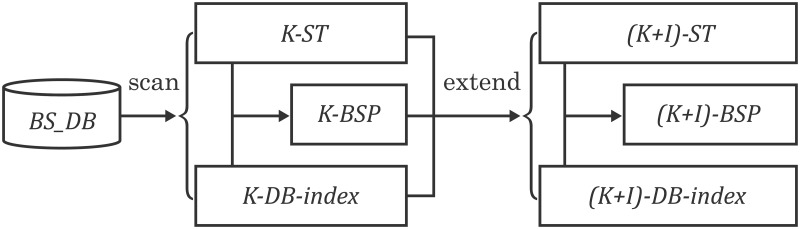
Algorithm model.

The proposed algorithm to mine the pattern in the biological sequences has four steps. (1) scan the sequence database once to construct the position table 1-ST and database index 1-DB-Index of single items; then the fast support counting algorithm is used to get frequent sequence 1-BSP. (2)in order to expand (k+1)-BSP from k-BSP(k = 1, k∈Z^+^), we propose a sequence connection strategy named as BS-Ext to obtain (k+1)-BSP by connecting k-BSP. (3)the index table ST and database index DB-Index will be updated to (k+1)-ST and (k+1)-DB-Index. (4) The (k+1)-BSP is obtained by the support counting algorithm. The recursive processes will be terminated until (k+1)-DB-Index is empty. These processes are shown in Fig 1. Definition 6. Sequence extension strategy BS-Ext. There are two k-BSP_*s*_ BSP_1_ and BSP_2_. If BSP_1_ and BSP_2_ can be connected, two conditions must be satisfied as below.

(1)|DB-index(BSP_1_)∩DB-index(BSP_2_)|≥mincount, that is the length of database index of BSP_1_ and BSP_2_ are greater than or equal to the minimum support count.

(2) ST(BSP_1_)[index].pos+1 = ST(BSP_2_)[index].pos, where the index is the serial number of BSP1 and BSP_2_ whose indexes are the same and their positions must be adjacent.

By extending strategy, a large number of infrequent candidate patterns are reduced.

Definition 7. Sequences connection BS-Con. A (k+1)-BSP is obtained by connecting two k-BSP_*s*_. If there are two k-BSP_*s*_ BSP_1_ = {BS_11_, BS_12_,⋯, BS_1*k*_} and BSP_2_ = {BS_21_, BS_22_,⋯, BS_2*k*_} and they can be connected. The connection of BSP_1_ and BSP_2_ can be expressed as BSP_1_⊕BSP_2_ = {BS_11_, BS_12_,⋯, BS_1*k*_, BS_21_, BS_22_,⋯, BS_2*k*_}.

Property 2. There are two k-BSP_*s*_, BSP_1_ and BSP_2_. The support count of BSP_1_⊕ BSP_2_ Support(BSP_1_⊕BSP_2_)≤|DB-index(BSP_1_)⋂DB-index(BSP_2_)|.

Proof. From the definitions of BS-Con, support count and DB-index, two conditions can be obtained as follows:

(1)Let BSP_3_ = BSP_1_⊕BSP_2_, then Support (BSP_1_⊕BSP_2_) = |DB-index(BSP_3_)|;

(2)Since DB-index(BSP_3_)⊆(DB-index(BSP_1_)⋂DB-index(BSP_2_)), we can get the expression |DB-index(BSP_3_)|≤|DB-index(BSP_1_)⋂DB-index(BSP_2_)|.

Therefore, Support_count(BSP_1_⊕BSP_2_)≤DB-index(BSP_1_)⋂DB-index(BSP_2_)|.

## Build ST and DB-index

The subsequences can be obtained from scanning database. Constructing ST and DB-Index of a subsequence BS can be seen in algorithm 1.

Algorithms 1. Build-ST-DB-index(BS_db, BS)

Input: Biological sequence database BS_db, subsequence BS.

Output: Position table ST(BS_1_) of subsequence BS_1_, database index DB-index(BS).

Begin:

(1) Set ST(BS) = Null, DB-index(BS) = Null;

(2) For each sequence BS_1_∈BS_db, Do

(3)  int i = 0;

(4)  If BS⊙BS_1_, then

(5)   SP_*BS*_*i*_ = POS(BS⊙BS_1_);

(6)   If SP_*BS*_*i*_.size>0,then

(7)    DB-index(BS).add(i);;

(8)   End if

(9)  End if

(10)  i++;

(11) End for

End

As can be seen from the algorithm 1, line (1) initializes the position table and the index database. Lines from (2) to (11) are the building processes, in which line (2) is a process of scanning databases once, line (5) finds all positions of BS appearing in BS_1_. If the BS appears in BS_1_, the index number of BS_1_ in the database will be added to the DB-index in line (7).

Using Algorithm 1, we can first find the position table for all single items in the sequence database. And then the sequence extension strategy BS-Ext and ST and DB-Index updating algorithm will be used to obtain an expanded sequence.

## Fast support counting and updating ST and DB-index

In this algorithm, the speed of computing support of sequence is a key factor to improve the efficiency of a sequence pattern mining algorithm. The pattern expansion technology is used to get the longer patterns. A new method of filtering out discontinuous patterns to prune and reduce unnecessary candidate patterns is described.

The algorithm is abbreviated as BSP-Distinguish.

Algorithms 2. Fast pattern recognition algorithm Distinguish (BS,minsup).

Input: Position table ST(BS), minimum support threshold minsup.

Output: Frequent biological sequence BSP.

Begin:

(1) int i = 0;

(2) For each position list SP(BS) in ST(BS), Do

(3)  If SP(BS) ≠ *ϕ*, then

(4)   i++;

(5)  End If

(6) End for

(7) If i/|ST(BS)|≥minsup, then

(8)  output BS as a BSP

(9) End If

End

As can be seen from Algorithm 2, line (1) initializes support count, line (2) scans position table ST(BS), the non-empty SP(BS) is counted, and the support count is the number of non-empty SP(BS). By comparing with minsup, the BSP is obtained inline (8).

When we use the fast support counting to get 1-BSP from 1-ST, it is important to use the sequence extension BS-Ext to obtain the longer sequences. Details of this can be seen in Algorithm 3.

Algorithms 3. ST-Update(k-ST,k-DB-index,k-BSPList,mincount).

Input: k-ST, k -DB-index, k-BSP set k-BSPList, minimum support count mincount.

Output: (k+1)-ST,(k+1)-DB-index.

Begin:

(1) For each k-BSP BSP_1_ in k-BSPList, Do

(2)  For each k-BSP BSP_2_ in k-BSPList, Do

(3)   DB-index = DB-index(BSP1)⋂DB-index(BSP_2_)

(4)   If |DB-index|≥mincount, then

(5)    For each index in DB-index, Do

(6)     If ST(BSP_1_)[index].pos+1 = ST(BSP_2_)[index].pos, then

(7)      BS_Ext = BSP_1_⊕BSP_2_

(8)      (k+1)-ST,(k+1)-DB-index = Build-ST-DB-index(DB-index, BS_Ext)

(9)     End If

(10)    End for

(11)   End If

(12)  End for

(13) End for

End

In the Algorithm 3, line (1) and line (2) traverse the k-BSP. According to line (3), the same database indexes of BSP_1_ and BSP_2_ are obtained. The number of the same indexes determines whether BSP_1_ and BSP_2_ satisfy the sequence extension strategy. In this step, the number of candidate patterns are reduced. Line (5) scans the database index DB-index. Line (6) determines whether the positions of BSP_1_ and BSP_2_ are adjacent to be connected. If BSP_1_ and BSP_2_ satisfy the extension conditions, Build-index-ST-DB(DB-index, BS_Ext) algorithm will be executed to acquire (k+1)-ST and (k+1)-DB-index after their connection. It is worth to notice that only database indexes are scanned in the process rather than the entire databases, which significantly improves the efficiency of the algorithm.

## Biological sequence pattern mining

The biological sequence pattern mining algorithm Mpbsmi contains building position table ST and database index DB-index, the algorithm of frequent pattern recognition and updating ST and DB-index. The specific processes can be seen in Algorithm 4.

Algorithms 4: Biological Sequence mining algorithm Mpbsmi.

Input: Biological sequence database BS_db, minimum support minsup.

Output: Frequent pattern set BSPList.

Begin:

(1) Set SingleItemList = *ϕ*;

(2) For each BS-DB, Do

(3)  For each item item in BS, Do

(4)   SingleItemList.add(item)

(5)  End for

(6) End for

(7) For each item item in SingleItemList, Do

(8)  {(1-ST,1-DB-index} = Build-ST-DB-index(BS-DB, item)

(9)  BSP-Distinguish(item, minsup)

(10) End for

(11) While(k>1&&|k-DB-index|≥0),Do

(12)  ST-Update(k-ST, k-DB-index, k-BSPList, |BS-DB|×minsup)

(13)  BSP-Distinguish(k-BS, minsup)

(14) End While

End

In the Algorithm 4, line (1) to line (6) is the process of scanning the database to obtain all the single items. The lines from (7) to (10) get the 1-ST and1-DB-index, and then 1-BSP is found. Lines from (11) to (14) update the ST and DB-index by the sequence extension, and obtain longer frequent patterns. The algorithm is explained further in below example 4.

Example 4. In order to mine the biological sequence patterns from the biological sequence databases in Table 1, we assume minsup = 50%. The steps are as follows.

(1)Scan the database to obtain the 1-BS as <I>, <F>, ……, <H>.

(2)Get frequent 1-ST and 1-DB-index as 1-ST = {<A> = [[11, 13, 28, 36, 39, 40, 45, 58, 89, 92, 99, 108, 127, 151], ……, [38, 46, 122, 167]]}, 1-DB-index = {<A> = [0, 1, 2, 3], ……, <Y> = [0, 1, 2, 3]}.

(3)Compute support of 1-BS from 1-ST and 1-DB-index. We get Support(I) = Support(F) = …… = Support(H) = 4/4 = 100%>50%. Therefore 1-BSP of <I>, <F>,⋯, <H> are obtained.

(4)The ST and DB-index will be updated by sequence extension. At last we get the ST and DB-index of the longer BSP as ST = {<TGITVNAVCPG> = [[153], [153], [153]], <PYSASKHGVVG> = [[131], [131], [131]]}, DB-index = {<TGITVNAVCPG> = [1, 2, 3], <PYSASKHGVVG> = [1, 2, 3]}. Then totally 263 BSP of <YSASKHGVV>, <YSASKHGVVG> etc. are obtained.

## Experiment

### Experimental set-up and data sources

The experiment was conducted on the platform running Windows 10 operating system with 8GB RAM, CPU of E5200@2.5GHz. The proposed algorithm has been compared with MSPM [27] and FBSB [28]. All the algorithms are written in java. The experimental data of protein sequences come from protein database Pfam 31.0 [33]. The datasets contains 12 protein families, where 3000 sequences are selected and tested as shown in Table 4. The data of the algorithm scalability for data size are shown in Table 5. The data of the algorithm scalability for data length are shown in Table 6.

**Table 4:** Experimental data of algorithm execution efficiency.

Proteinfamily	Identification number	Total sequences	Average length	Test sequences
*Adh*_*short*	PF00106	86592	180.90	200
*Lectin*_*legB*	PF00139	3578	219.20	278
*Glyco*_*hydro*_19	PF00182	2230	152.50	327
*G* − *alpha*	PF00503	6158	307.00	380
*Acetyltransf*_1	PF00182	63111	121.30	223
*TatC*	PF00902	3594	212.50	210
*ZnuA*	PF01297	5307	273.40	300
*Metalloenzyme*	PF01676	5899	425.50	280
*Peptidase*_*S*66	PF02016	2533	283.00	260
*TSP*_3	PF02412	6644	31.70	300
2*OG* − *FeII*_*Oxy*	PF03171	14598	104.80	135
*Efhand*_3	PF09069	1080	90.30	107

**Table 5:** Experimental data of algorithm scalability on data size.

Proteinfamily	Identification number	Total sequences	Average length	Test sequences
*Cactin*_*mid*	PF10312	782	180.20	600
*EF* − *hand*_3	PF09069	1080	90.30	600
*Cation*_*ATPase*_*C*	PF00689	13024	180.10	600

**Table 6:** Experimental data of algorithm scalability on data length.

Proteinfamily	Identification number	Total	Average length	Test sequences	Groups
*Abhydrolase*_4	PF08386	2515	100.60	200	2
*Cthe*_2159	PF14262	678	200.50	200	2
*Choline*_*transpo*	PF04515	2596	300.00	200	2
*ABC*_*ATPase*	PF09818	552	403.80	200	2

### Performance evaluation of algorithm

Experiment 1. Time cost analysis of the relationship between support threshold and time consumption.

**Fig 2:**
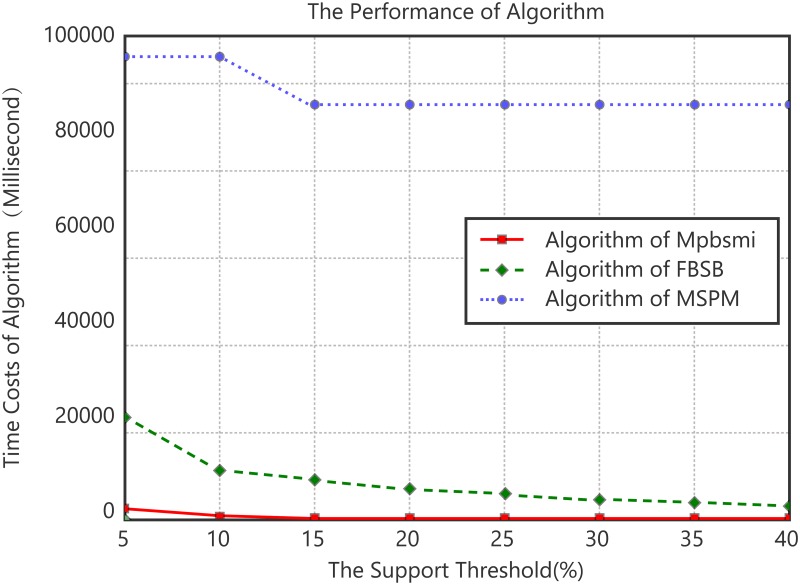
Algorithm efficiency comparison.

The experiment of the relationship between the support threshold and the time cost was carried out by group experiment. The 3000 sequences were divided into 30 groups in average, in which the average length of sequence is 200.175. The support threshold changes from 5% to 40%. The results are the average values of 30 groups. The experimental results are shown in Fig 2. Although as depicted in Fig 2, the time costs of the algorithm Mpbsmi, FBSB and MSPM all decrease with the increase of the support threshold, the time consumed by the proposed algorithm Mpbsmi is significantly lower than FBSB and MSPM algorithms. The execution time of FBSB algorithm is around 10 times that of Mpbsmi algorithm. The main reason is that the Mpbsmi algorithm only processes sequence database index but the FBSB algorithm scans the entire sequence database. Meanwhile the time consumption of MSPM algorithm is significantly higher than Mpbsmi and FBSB algorithm, which is because MSPM algorithm produces a large number of basic candidate pattern s, sorting and combining the pattern is time-consuming. Mpbsmi reduces a large number of candidate patterns by sequence extension strategy BS-Ext, which reduces time cost.

Experiment 2. Algorithm scalability analysis on data size of the relationship between data size and time consumption.

**Fig 3:**
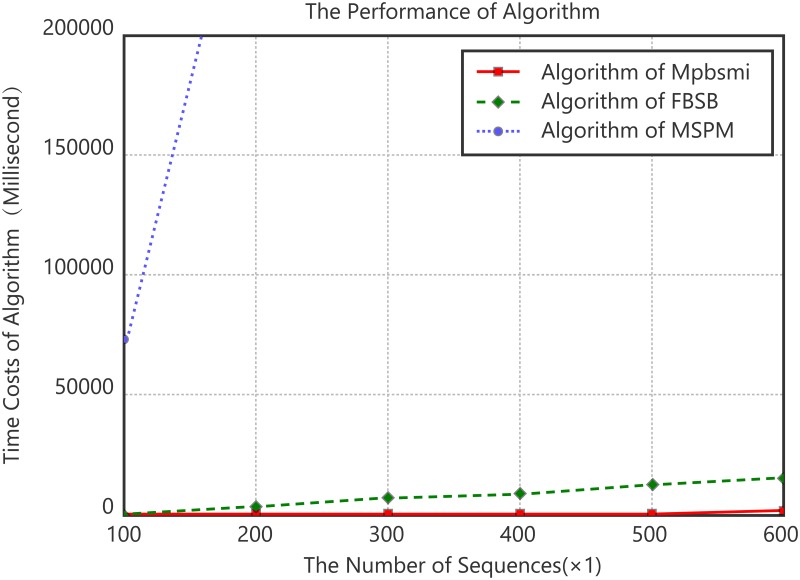
Algorithm scalability comparison of data size.

The data used in the experiment is shown as Table 5. Every experimental group contains one protein family and selects 100, 200, 300, 400, 500 and 600 sequences respectively. The support threshold is set to 40%. The results are the averaged values of three groups. The experimental results are shown in Fig 3. As illustrated in Fig 3, the time consumption of algorithm MSPM rises linearly, and the time cost lines of the algorithm FBSB and Mpbsmi rise slowly. At the same time, the efficiency of Mpbsmi algorithm is noticeably higher than that of MSPM and FBSB, which indicates that Mpbsmi algorithm has better scalability of data size.

Experiment 3. Algorithm scalability analysis on data length of the relationship between average sequence length and time consumption.

**Fig 4:**
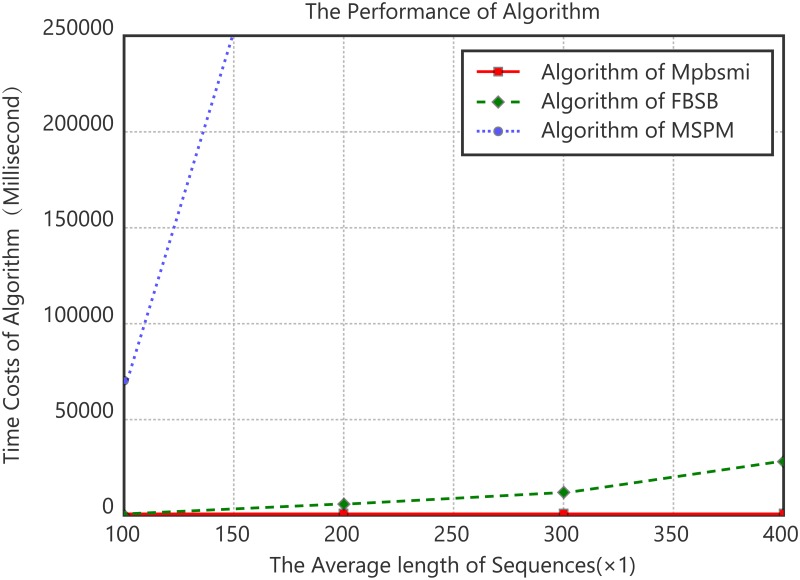
Algorithm scalability comparison of data length.

The experimental data are four protein families in Table 6. The average length of sequences changes from 100 to 400. Every protein family data are divided into two groups and the groups select 100 and 200 sequences respectively. The experimental results use the average values of every two groups. The support threshold is set to 40%, the experimental results are shown in Fig 4.

As can be seen from Fig 4, with the average length of the sequence increasing, time consumption of the algorithm continues to increase. At the same time, the time costs of Mpbsmi are significantly lower than that of FBSB and MSPM. It is shown that the Mpbsmi algorithm has a good scalability in respect to data length.

Experiment 4. The mining results of algorithm.

In the case of a fixed support threshold of 40% with the same as Experiment 2, the result of the biological sequence patterns obtained are shown in Table 7: the first column shows the support threshold, the (k+1)^*th*^ column and the k^*th*^ column are the data set size and the corresponding number of pattern is mined, wherein 1<k<7 and k∈Z^+^. As shown in the table, with the increment of the data set, the number of sequence pattern varies between 124 and 173, which reflects that the total single items of the biological sequences is constant with long sequence. Under the different data sets, if the support threshold is constant, the number of sequence patterns is relatively stable. When the support threshold is changed from 5% to 40% with the same data used in Experiment 1. The mining results are shown in Table 8: as the support threshold increases, the number of sequence patterns is gradually reduced. In this experiment, we obtain all the continuous patterns. Because the algorithm can mine all successive sequence patterns, the algorithm’s accuracy is 100% with no errors.

**Table 7:** Sequence patterns under different size of data sets.

Supportthreshold	BSP	Data size	BSP	Data size	BSP	Data size
40%	170	100	171	300	171	500
40%	170	100	171	300	171	500
40%	135	100	138	300	139	500
40%	171	200	172	400	173	600
40%	144	200	146	400	124	600
40%	133	200	137	400	136	600

**Table 8:** Table caption Nulla mi mi, venenatis sed ipsum varius, volutpat euismod diam.

Supportthreshold	BSP	Support threshold	BSP
5%	3198	25%	304
10%	1037	30%	233
15%	625	35%	192
20%	413	40%	164

Experiment 5. The memory and CPU usage analysis.

In the case of a fixed support threshold of 40% with the same experimental data used in Experiment 2, the memories and CPU used by the algorithm to process the data are shown in Table 9. The results are the average of three times running data. As one can see from Table 9, the peak memory usage by Mpbsmi algorithm is closer to that of the FBSB algorithm. Similarly, Mpbsmi and FBSB algorithms have the similar CPU peak values. The memory peak of MSPM algorithm is relatively higher than that of the other two algorithms but CPU peak is lower. It can be seen that Mpsmi and FBSB have lower memory and higher CPU utilization than MSPM algorithm, which improves the efficiency of algorithm and reduces the time consumption by using high CPU utilization.

**Table 9:** The memory usage of algorithms.

Algorithms	Peak value of memory	Peak value of CPU occupancy ratio	Support threshold
*Mpbsmi*	549.6MB	54.6%	40%
*FBSB*	553.3MB	53.6%	40%
*MSPM*	602.8MB	49.6%	40%

The experimental results are used to calculate the P-value between the algorithm Mpbsmi and the FBSB, the algorithm Mpbsmi and the MSPM. The smaller the P- value, the more reason to think that the difference between things is a statistical indicator. As can be seen from Table 10, there are significant statistical differences in the efficiency difference of algorithm execution.

**Table 10:** P-value: Analysis of efficiency difference of algorithm.

Experiments	Mpbsmi and FBSB	Mpbsmi and MSPM
Experiment 1	0.01378	6.6974*10^−11^
Experiment 2	0.00006	0.002
Experiment 3	0.035	0.044

## Discussion

In this paper, a new biological sequence pattern mining algorithm, the Mpbsmi, based on database index technique is presented. Compared to the two recently reported algorithms MSPM and FBSB, our proposed Mpbsmi algorithm uses a position table abbreviated ST and sequence index structure DB-Index with specially designed sequence pattern expansion and fast support counting method. Whilst the MSPM is an algorithm based on prefix tree and pattern extension, and FBSB is an algorithm based on bitmap structure and a quick sort list. By using index technology, our algorithm has shown significant performance improvement.

(1)Traditional frequent pattern mining algorithms

The traditional frequent pattern algorithms [6–21] mentioned earlier in this paper are transaction based. They are designed for short sequences process with limited capability of processing long sequences. The biological sequences are characterized as ordered, continuous and elements repetitive. These features have constraint the efficiency of traditional algorithms. In terms of the internal data structures used by these algorithms, tables or tree structure are mostly used. The efficiency of query on large scale database is typically low. Literatures [23–25] have taken advantage of using the memory index as internal data structure, however they are vulnerable to memory overflow when dealing with longer sequences without sequence pattern restricting.

(2) Biological sequence pattern mining algorithm.

The algorithms [26–28] mentioned in the paper are biological sequence pattern mining algorithms. The BioPM [26] algorithm is based on the prefix projection technology, MSPM [27] algorithm is based on prefix tree. They are both developed based on traditional sequential pattern technologies. The MSPM has shown higher efficiency than that of BioPM. FBSB [28] utilizes the bitmap as data structure and use this structure to calculate the support level. It has shown that FBSB algorithm has over performed MSPM algorithm in terms of efficiency.

(3) The algorithms based on the parallel computing technology.

Another co-existing biological sequence mining algorithm stream is to take advantage of parallel computing. Literatures [29–32] used the MapReduce and Spark framework based on distributed statistical approach and are suitable for cloud computing environment running on multiple computers.

The proposed algorithm in this paper has proved its merit to obtain continuous biological sequence patterns efficiently. By further improvement, it can be used to acquire all the biological sequence patterns. There is also a plan to adapt the algorithm in cloud computing environment.

## Conclusion

This paper proposes a new algorithm Mpbsmi based on data index technology for improving the efficiency of biological sequence pattern mining. Based on the index technique, a sequence position table ST is proposed to record the position information of sequences and subsequences. At the same time, the database index of sequences and subsequences is established as DB-index. The ST and DB-index are scanned only when the sequence pattern is extended. Through the sequence extension strategy and the position table, the algorithm achieves fast patterns mining and filters out a large number of invalid candidate patterns to get the entire continuous sequence patterns. Finally, the experimental results show that the proposed algorithm is superior to the existing biological sequence pattern mining algorithm such as MSPM and FBSB in terms of efficiency and scalability.
